# Genetic Diversity Analysis and Core Germplasm Collection Construction of Tartary Buckwheat Based on SSR Markers

**DOI:** 10.3390/plants14050771

**Published:** 2025-03-03

**Authors:** Yuanzhi Cheng, Jing Zhang, Ziyang Liu, Bin Ran, Jiao Deng, Juan Huang, Liwei Zhu, Taoxiong Shi, Hongyou Li, Qingfu Chen

**Affiliations:** 1 Research Center of Buckwheat Industry Technology, School of Life Sciences, Guizhou Normal University, Guiyang 550025, China; 18886024163@163.com (Y.C.); 18886063160@163.com (J.Z.); feblzyy@163.com (Z.L.); ranbin526478090@163.com (B.R.); ddj613@163.com (J.D.); huang200699@163.com (J.H.); liweib0401001@163.com (L.Z.); shitaoxiong@gznu.edu.cn (T.S.); 2 Guizhou Key Laboratory of Biotechnology Breeding for Special Minor Cereals, Guiyang 550006, China

**Keywords:** Tartary buckwheat, genetic diversity, population structure, core germplasm collection, SSR

## Abstract

Tartary buckwheat is an important medicinal and edible crop known for its significant health benefits to humans. While numerous Tartary buckwheat germplasm resources have been collected in China, the genetic diversity and core germplasm resources remain largely unclear. The aim of this work was to analyze the genetic variability and construct a core germplasm collection of Tartary buckwheat. Fifteen highly polymorphic SSR markers were used to investigate 659 Tartary buckwheat accessions. A total of 142 alleles were marked, with an average of 9.47 alleles per locus. Genetic variability analysis revealed that these collected accessions exhibit high genetic diversity and can be classified into seven subgroups. Among wild, landrace, and improved accessions, the wild accession showed the highest genetic diversity, while no significant genetic variation was observed between the landrace and improved accessions. Based on genetic diversity and population structure analyses, a core germplasm collection containing 165 accessions (47 wild, 92 landrace, and 26 improved) was constructed, ensuring high genetic diversity and good representation. This study not only highlighted the genetic differences among Tartary buckwheat accessions, but also provided insights into the population structure and the development of a core germplasm collection. It provided important references for the conservation of genetic diversity and the genetic improvement of Tartary buckwheat.

## 1. Introduction

Tartary buckwheat (*Fagopyrum tataricum* Gaertn.), a member of the *Fagopyrum genus* in the *Polygonaceae* family, is a pseudocereal. It most probably originated from southwest China and is widely cultivated in Asia, Europe, and North America [1,2,3]. Tartary buckwheat grain is not only rich in starch, proteins with a balanced composition of essential amino acids (methionine, tryptophan, lysine, histidine), lipids, and minerals, but also contains abundant secondary metabolites such as flavonoids, phenolic acids, and triterpenoids [1,4]. A large number of studies have suggested that Tartary buckwheat grain confers various health benefits to humans, including anti-oxidants, anti-inflammatories, anti-diabetic, anti-obesity, anti-hypertensives, and anti-carcinogenic effects, due to its high content of nutrients and bioactive secondary metabolites [4,5,6]. As a result, Tartary buckwheat is considered a promising smart food crop for the future, and its grains have been used to develop TB powder, TB capsule, TB alcohol, TB noodle, TB tea, TB vinegar, and TB bread [1,6].

Genetic resources are the foundation of breeding progress. However, Tartary buckwheat breeding has progressed slowly over the past two decades, mainly due to the limited genetic diversity information available, which has greatly restricted the genetic improvement of the crop. Therefore, research on the genetic diversity of Tartary buckwheat, especially in large-scale germplasm collections, is urgently needed.

Morphological, cytological, and molecular marker technologies are the major methods for studying genetic diversity [7]. Generally, morphological analysis can reveal genetic variation in genetic resources to a certain extent, but it is easily affected by environmental factors. Cytological analysis is time-consuming and labor-intensive. In contrast, molecular marker technology is simpler, and the results are more stable. Over the past several decades, many DNA-based molecular markers, including random amplified polymorphic DNA (RAPD) [8], insertion/deletion (InDel) [9], simple sequence repeat (SSR) [10], and single-nucleotide polymorphism (SNP) [11], have been used for genetic analysis in many plants [12,13,14,15]. Among these markers, SSRs are more consistent than RAPD, more polymorphic than ISSRs, and easier to genotype than SNPs, making them widely used for genetic diversity analysis in plants [16,17,18].

To date, several studies have explored the genetic diversity of Tartary buckwheat based on SSR markers. Sabreena et al. used seven ISSR and seven SSR markers to investigate the diversity of 35 Tartary buckwheat genotypes, demonstrating that both marker techniques are highly effective for genetic diversity assessment in Tartary buckwheat [19]. Song et al. carried out a genetic diversity analysis of 112 Tartary buckwheat accessions from 29 populations using 10 SSR markers, revealing a high level of genetic diversity and significant genetic structure differentiation among the accessions [2]. Kishore et al. found that 71 Tartary buckwheat populations exhibited a broad genetic base using seven SSR markers [20]. More recently, Pipan et al. [21] and Balážová et al. [1] also used 24 and 21 SSR markers, respectively, to explore the genetic diversity of Tartary buckwheat, concluding that SSR marker technology is a reliable and stable technique for genetic diversity analysis and high genetic diversity exists in the Tartary buckwheat germplasms. Although these studies have provided insights into the genetic diversity of Tartary buckwheat germplasms, they have been limited by the small number of germplasm samples. More importantly, there is no or less investigation into the genetic diversity among wild, landrace, and improved Tartary buckwheat accessions in these studies.

In the present study, we analyzed the genetic diversity and population structure of 659 Tartary buckwheat accessions, including 101 wild, 383 landrace, and 175 improved accessions (breeding line and varieties), using 15 SSR markers. The research aimed to assess population variability and construct a core germplasm collection of Tartary buckwheat across different ecological conditions. The results not only provide valuable information for the selection and breeding of Tartary buckwheat germplasm resources but also offer a foundation for fully exploring and utilizing the excellent Tartary buckwheat genetic resources and formulating new hybrid combinations in the future.

## 2. Results

### 2.1. SSR Polymorphism and Allelic Diversity

All 15 selected SSR loci were polymorphic, and a total of 142 alleles were amplified in the 659 Tartary buckwheat accessions (Table 1 and Table S1). The number of alleles (Na) per locus ranged from 4 (SSRT33 and SSRT39) to 18 (P128), with an average of 9.47; the average number of effective alleles (Ne) per locus was 1.931, ranging from 1.018 (SSRT26) to 3.406 (Ft1ssR21) (Table 1). The observed heterozygosity (Ho) ranged from 0.03 (SWUFt169) to 0.982 (TatG0200), with an average of 0.224; the expected heterozygosity (He) ranged from 0.018 (SSRT26) to 0.706 (Ft1ssR21), and its average was 0.62. The average Shannon information index (I) was 0.895, ranging from 0.072 (SSRT26) to 1.568 (Ft1ssR21). The polymorphism information content (PIC) value ranged from 0.018 (SSRT26) to 0.651 (Ft5ssR20), with an average of 0.391. For the inbreeding coefficient (Fis), the primers SSR23 and TatG0200 exhibited the highest (0.932) and lowest (−0.616) values, respectively, and the average Fis was 0.498.

### 2.2. Genetic Diversity Among Wild, Landrace, and Improved Tartary Buckwheat Accessions

The genetic diversity parameters, including Na, Ne, Ho, He, and I for the wild, landrace, and improved accessions, are presented in Table 2. All 15 selected SSR loci were polymorphic in each type of accession. Among the three type accessions, the average of Na was landrace accession (7.667) > wild accession (7.133) > improved accession (4.40), and the average of Ho value was landrace accession (0.228) > wild accession (0.225) > improved accession (0.216). In addition, the average of Ne was wild accession (3.324) > landrace accession (1.748) > improved accession (1.657), the average of uHe value was wild accession (0.591) > landrace accession (0.369) > improved accession (0.326), and the average of I value was wild accession (1.235) > landrace accession (0.731) > improved accession (0.605). The average of Ne, uHe, and I in the wild accession were significantly higher than those in the landrace and improved accessions, without obvious differences observed between the landrace and improved accession (Table 2).

**Figure 1:**
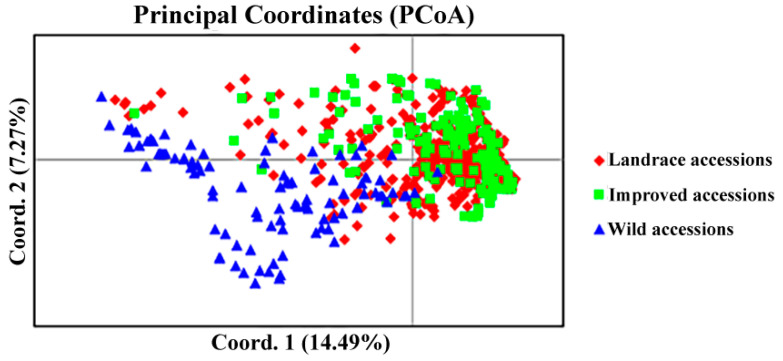
Distribution of principal coordinates of the landrace, improved, and wild accessions in all 659 Tartary buckwheat accessions. Notes: red rhombus, green square, and blue triangle represent the landrace accession, improved accession, and wild accession, respectively.

Genetic distance and genetic identity analysis among wild, landrace, and improved accessions also revealed a similar relationship, with the wild accession exhibiting greater genetic distance and lower genetic identity compared to the landrace and improved accessions. On the contrary, no significant difference was observed between landrace accession and improved accession. These findings indicate that the genetic diversity of the wild accession is obviously higher than that of the landrace and improved accessions, while the genetic variation between the landrace and improved accessions is relatively narrow (Table 3). The genetic diversity among wild, landrace, and improved accessions was further confirmed by PCoA analysis (Figure 1). Analysis of gene flow and genetic differentiation coefficient revealed significant gene exchange between landrace and improved accessions, while the gene exchange between wild accession and either landrace or improved accessions was considerably lower than that between landrace and improved accessions (Table 4).

### 2.3. Genetic Similarity Coefficient Analysis and Cluster Analysis

Wide genetic variations were observed among the 659 Tartary buckwheat accessions, although no significant genetic diversity was detected among a few varieties (Tables S2 and S3). The genetic distance among the accessions ranged from 0 to 3.496.

**Figure 2:**
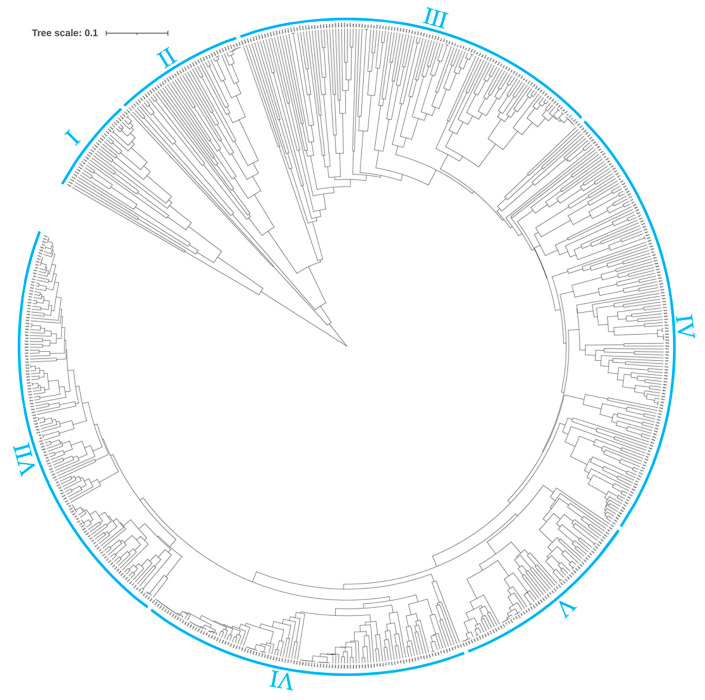
Clustering of 659 Tartary buckwheat accessions based on 15 SSR markers using UPGMA.

Based on the genetic distance matrix of the 659 Tartary buckwheat accessions, clustering analysis was carried out using the UPGMA method, and a phylogenetic tree was constructed (Figure 2). The 659 Tartary buckwheat germplasm resources were divided into seven subgroups, consisting of 32 (32 wild), 45 (41 wild and 4 landrace), 123 (27 wild, 66 landrace and 30 improved), 146 (115 landrace and 31 improved), 66 (41 landrace and 25 improved), 110 (57 landrace and 53 improved), and 137 (1 wild, 100 landrace and 36 improved) accessions, respectively (Figure 2 and Table S4). The genetic distance of subgroups I and II was significantly higher than the other five subgroups. Notably, 100% and 91% of the members in subgroups I and II were wild accessions. Additionally, the landrace and improved accessions were clustered together, as shown in subgroups IV, V, VI, and VII. These suggested that the genetic diversity of wild accessions was higher than landrace and improved accessions. Interestingly, subgroup III was the only cluster that included the wild (27), landrace (66), and improved (30) accessions (Figure 2 and Table S4), indicating that this subgroup might be a transitional subgroup between wild Tartary buckwheat and Tartary buckwheat. Notably, all the 30 improved accessions in subgroup III were bred from the landrace accessions by the systemic breeding approach, which was consistent with their clustering alongside both wild and landrace accessions (Table S4).

### 2.4. Analysis of Population Structure

**Figure 3:**
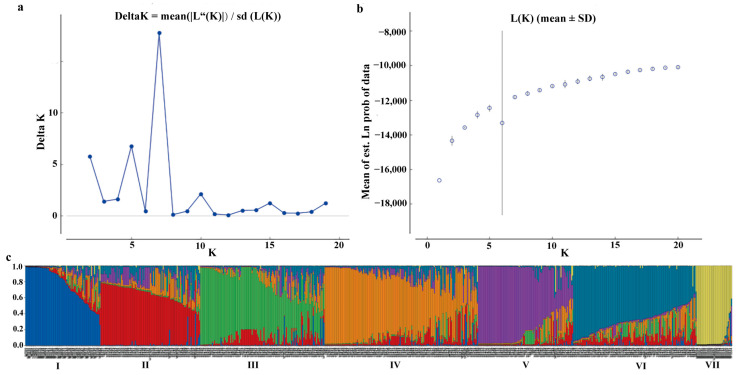
Population structure of 659 Tartary buckwheat accessions based on SSR analysis. (**a**) Estimation of the optimum number of subgroups (K). The maximum value of ∆K at K = 7 suggests seven subpopulations. (**b**) Graph for the parameter LnP (D) and each value of K. (**c**) Population structure when K = 7. The proportion of each color indicates the probability of each accession being divided into the corresponding subgroup.

To further explore the genetic structure of the 659 Tartary buckwheat accessions, the optimal value K was calculated. As a result, a clear peak for Delta K was observed at K = 7 in the plots of L (K) versus Delta (Figure 3a,b), indicating that these Tartary buckwheat accessions could be divided into seven different groups (Figure 3c). The seven groups are represented by different colors, corresponding to subgroups I, II, III, IV, V, VI, and VII, with subgroup IV containing the highest number of accessions (143). Notably, all members of subgroup VII (33) and the vast majority of members of subgroup I (55) belong to wild Tartary buckwheat accessions, which were collected from the Tibet province in China (Table S1). Subgroup I also contained 14 landrace accessions (7 from Tibet, 4 from Sichuan, and 3 from Yunnan) and one improved accession (from Guizhou), in addition to the 55 wild accessions. The highest numbers of landrace accessions (98) and improved accessions (47) were found in subgroups IV and V, respectively (Table S1). All subgroups, except for subgroup VII, contained both southwestern landraces and northern landraces in China. Furthermore, there is significant gene exchange between different subgroups, and some members of subgroups II, III, and IV presented a complex genetic background (Figure 3c).

### 2.5. Construction of Core Collections

**Figure 4:**
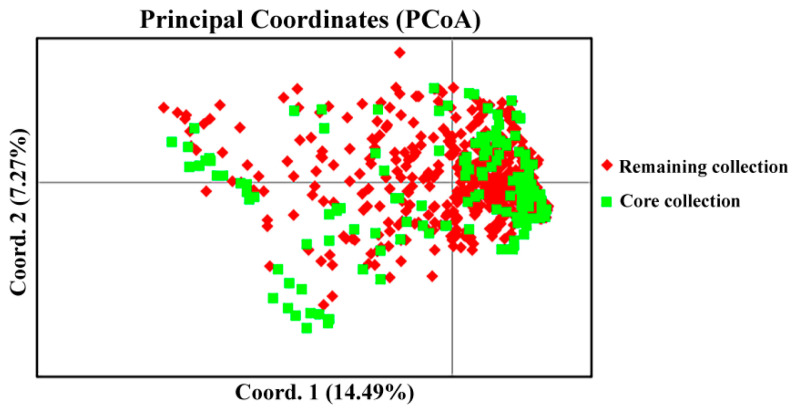
Distribution of principal coordinates of the core collection in all collections. Note: Red rhombus and green square represent the remaining collection and core collection, respectively.

Core collections were constructed using corehunter3.0 software. Based on the criterion that the remaining percentage of polymorphic loci was >80%, core collections were extracted in sizes of 30%, 25%, 15%, 10%, and 8% of all collections, respectively (Table 5). As shown in Table 5, the core germplasm collection exhibited higher values for Ne, Ho, He, and I compared to the original collection. The *t*-test analysis revealed that only the 30% pre-core collection and the 25% pre-core collection showed no significant differences in the Na, Ne, Ho, uHe, and I values compared with the original collection. According to the principle of minimum germplasm number, the 25% pre-core collection was selected to construct the core germplasm collection, which consisted of 165 Tartary buckwheat accessions, including 47 wild, 92 landrace, and 26 improved accessions (Table S5). Genetic diversity analysis of the remaining 494 Tartary buckwheat accessions indicated that there was also no obvious difference compared with all 659 Tartary buckwheat accessions (Table 6). In addition, principal coordinate analysis of 659 Tartary buckwheat germplasm resources showed that the core germplasm collection was evenly distributed within the entire collection (Figure 4). This suggests that the constructed core germplasm collection was highly representative.

## 3. Discussion

Understanding the genetic relationship among a large-scale collection of Tartary buckwheat accessions is crucial for the germplasm innovation and breeding of new cultivars [22,23]. Molecular markers have been widely used to investigate genetic variations at the DNA level [8,9,10,11,12,13,14,15,24]. Among all molecular markers, SSR markers are the most frequently used in genetic diversity analysis due to their codominant inheritance and rich polymorphism. More importantly, the integration of SSR technology with capillary fluorescence electrophoresis has significantly improved the safety, accuracy, and efficiency of genetic analysis, reducing the error rate associated with manual interpretation of results compared to traditional polyacrylamide gel electrophoresis [2,24,25]. In this study, 142 alleles were identified in 659 Tartary buckwheat accessions using 15 SSR markers, with an average of 9.47 alleles in each locus. The number of alleles observed per SSR maker was obviously higher than those reported in previous studies [2,19,20], suggesting that the SSR markers employed in our study were highly polymorphic. Among the 15 SSR markers, the PIC value ranged from 0.018 to 0.651, and the average was 0.391, which was lower than the values reported by Song [2] and Li [26]. The reason might be the larger number of Tartary buckwheat accessions in our study, some of which might exhibit a higher degree of homogenization. In fact, a few germplasms could not be distinguished by 15 pairs of SSR primers in our analysis. The genetic distance in 659 Tartary buckwheat accessions ranged from 0 to 3.496, and the mean I of 0.895 indicated that these Tartary buckwheat germplasm resources had abundant genetic diversity.

Exploring the genetic relationship among wild, landrace, and improved accessions would be crucial for fully utilizing the excellent Tartary buckwheat genetic resources to breed new varieties by formulating new hybrid combinations. In this study, we analyzed the genetic relationship among wild (101), landrace (383), and improved (175) Tartary buckwheat accessions. The I of wild accessions was significantly higher than those for landrace and improved accessions, while no significant differences were observed between the landrace and improved accessions. Similarly, the genetic distance between wild and landrace accessions (0.256) and between wild and improved accessions 0.281) was obviously notably larger than the distance between landrace and improved accessions (0.006). These findings indicate that the genetic diversity of wild accession is much higher than that of landrace and improved accessions, while there is no significant difference in genetic diversity between landrace and improved accessions. This is consistent with the previous study by Zhang et al., which identified notable genetic divergence between wild and cultivated accessions based on SNP markers analysis [3,27]. Furthermore, the results of population structure analysis, cluster analysis, and genetic identity analysis among wild, landrace, and improved accessions further support the difference in genetic diversity among these three accessions. Gene flow analysis found that significant gene exchange happened between landrace and improved accessions, while a lower gene exchange existed between wild and either landrace or improved accessions, respectively. This is consistent with the observation that wild accession has higher genetic diversity than that of landrace and improved accessions, and there is lower genetic variation between landrace and improved accessions. The lower genetic diversity between landrace and improved accessions may be closely related to the breeding technology where systemic breeding or Tartary buckwheat was the major approach, resulting in many breeding varieties directly derived from landrace varieties [28]. Notably, in this study, the 659 Tartary buckwheat accessions were divided into seven subgroups. Interestingly, among the seven subgroups, we found that subgroup VII and subgroup I exhibited the largest genetic distance and genetic diversity. All members of subgroup VII belonged to wild Tartary buckwheat accessions. Subgroup I consisted of 14 landrace accessions (7 from Tibet, 4 from Sichuan, and 3 from Yunnan) and one improved accession (derived through systemic breeding from a Guizhou landrace) except 55 wild accessions. All these findings indicated that Tibet, in China, is most likely the origin center of cultivated Tartary buckwheat, consistent with previous research [3,27]. Zhang et al. [3] and He et al. [27] proposed that the domestication of Tartary buckwheat occurred independently in the southwestern and northern regions of China. Interestingly, in our study, we found that all subgroups, except subgroup VII and subgroup I, contain both southwestern landraces and northern landraces from China. This result contrasts with the findings of Zhang et al. [3] and He et al. [27]. The discrepancy may be attributed to the limited number of molecular markers used in our study or differences in the materials examined. To address this question, further genetic diversity analysis based on whole-genome SNPs is needed.

The construction of a core germplasm collection is a crucial step in genetic research. Generally, the core germplasm collection could be constructed using morphological data and molecular marker data. However, the morphological data can be easily affected by the developmental stages and the environment. In contrast, molecular markers provide abundant genetic variation and their stability is not affected by these variables. Thus, they have been widely used in core germplasm collection construction [7,22,24,29,30]. Although several studies have performed the genetic diversity analysis of Tartary buckwheat using SSR markers [1,2,19,20,21,26], no core germplasm collection construction has yet been carried out until now. In this study, 165 germplasms (25.04%) from 659 accessions were selected to construct the core germplasm collection of Tartary buckwheat based on SSR markers, including 47 wild, 92 landrace, and 26 improved accessions. The 165 core accessions contained 92.25% (131/142) polymorphic loci of 15 SSR markers. The significance test among the core germplasm collection, the retention resources, and the original germplasm collection demonstrated that there were no significant genetic diversity differences. Furthermore, PCoA showed that core accessions were evenly distributed in the original accession. All these findings indicate that the 165 accessions selected as the core germplasm collection accurately represent the genetic diversity of the entire 659 accessions, and it can serve as the key resource for the diversity conservation. More importantly, it also can serve as the crucial resource for genetic improvement of Tartary buckwheat, which could be based on their genetic diversity, agronomic traits, quality traits, stress resistance traits. However, further investigation of these traits in the core germplasm is needed in future studies.

## 4. Materials and Methods

### 4.1. Plant Materials

A total of 659 Tartary buckwheat accessions were used in this study, including 101 wild, 383 landrace, and 175 improved accessions (Table S1). All these accessions were collected in 15 provinces in China and maintained in the Research Center of Buckwheat Industry Technology of Guizhou Normal University (Guiyang, Guizhou, China).

### 4.2. DNA Isolation and PCR Amplification

All accessions were planted in the experimental field (Anshun, Guizhou Province). Leaves from a single plant for each accession were collected at the six-leaf stage. Genomic DNA from the leaf of each Tartary buckwheat accession was extracted using a modified CTAB method [31]. The concentration and purity of the isolated DNA were evaluated using the NanoDrop 1000 ultraviolet spectrophotometer (Thermo Scientific, Wilmington, NC, USA). Forty-eight SSR primer pairs were used to perform PCR amplification and capillary electrophoresis on 10 Tartary buckwheat accessions with distinct phenotypic differences to screen for polymorphic primers. A total of 15 SSR primer pairs exhibited clear polymorphism and were selected to analyze the genetic diversity of 659 Tartary buckwheat accessions. Detailed information on these SSR primer pairs is provided in Table S6. All primers were purchased from Sangon Biotech Co., Ltd. For each pair primers, the fluorescent accession was added to the 5′end of the forward primer. The PCR amplification volume was 10 µL, containing 5 µL 2 × Taq PCR Master Mix (BioTeke Corporation, Wuxi, China), 0.5 µL of each primer, 1 µL template DNA (~20 ng), and 3 µL distilled water (ddH_2_O). For the negative control, the PCR amplification volume was 10 µL, containing 5 µL 2 × Taq PCR Master Mix (BioTeke Corporation, Wuxi, China), 0.5 µL of each primer, and 4 µL distilled water (ddH_2_O). The PCR was performed according to the following cycling parameters: initial denaturation for 5 min at 95 °C; then 35 cycles at 95 °C for 30 s, 52 °C–62 °C for 30 s, and 72 °C for 30 s, with a final extension at 72 °C for 20 min. For PCR amplification, two duplications were performed for each sample. The detection of amplification products was performed using capillary electrophoresis (CE) with a ABI 3730XL DNA Analyzer (Applied Biosystems, Waltham, MA, USA). In brief, the fluorescent PCR product was diluted to a uniform multiple and added to the upper plate. The sampling system was as follows: fluorescent PCR product 1 µL, GeneScan™500 LIZ (molecular weight internal standard [32], AppliedBiosystem) 0.5 µL, and Hi-Di™ Formamide (AppliedBiosystem) 0.5 µL. The mixture was subjected to 95 °C denaturation on PCR amplifier for 3 min. Then the PCR products were analyzed using an ABI 3730XL DNA Analyzer.

### 4.3. Genetic Diversity and Structure Analysis

Genetic diversity indicators were calculated using GenAlEx version 6.501 [33], including the number of different alleles (Na), number of effective alleles (Ne), Shannon diversity index (I), expected heterozygosity (He), and observed (Ho) heterozygosity. Gene flow (Nm) was calculated based on the genetic differentiation coefficient (Fst) obtained from GenAlEx version 6.501 [33]. Cervus version 3.0.7 software (Copyright Tristan Marshal, Field Genetic, Ltd., London, UK) was used to determine the polymorphic index content (PIC) locus [34]. Genetic distance was calculated using PowerMarker version 3.25 [35]. A phylogenetic tree was constructed based Nei’s genetic distance using the algorithm UPGMA (unweighted pair group method of arithmetic clustering) by MEGA version 7.0 [36]. Population structure analysis was carried out as described previously by DeBoer et al. [37] by using STRUCTURE 2.3.4 software [38]. The parameters used in the STRUCTURE analysis was follows: the K value set as 1 to 20, burn-in cycle set as 10,000, MCMC set as 100,000; run 20 times for each k value. The best △K value was calculated using the STRUCTURE HARVESTER version 0.6.93. The results of the structural analysis were drawn using the software Clump and Distract based on the best ΔK value.

### 4.4. Construction and Evaluation of the Core Germplasm Collection

Corehunter version 3.0 software [39] was used to extract the germplasm, with the sampling ratio set as 10% to 30% under default parameters. Genetic diversity indicators of the core germplasm, retention germplasm, and original germplasm were calculated using GenAlEx version 6.501. Differences in the genetic parameters between extract core germplasm and original germplasm as well as between retention germplasm and original germplasm were estimated using Excel under *t*-test. The core germplasm collection was considered when the extracted germplasms contained ≥80% polymorphic loci of 15 SSR markers and there was no significant difference in each genetic parameter among the extracted germplasm, retention germplasm, and original germplasm. Using PcoA analysis, the effectiveness of the core germplasm was evaluated based the distribution range of core resources in the original germplasm.

## 5. Conclusions

In this study, 15 SSR markers were used to assess the genetic diversity and population structure of 659 Tartary buckwheat accessions. The findings revealed that the 659 accessions exhibited a high level of genetic diversity and could be divided into seven accessions. Additionally, the wild accession showed abundant genetic diversity than landrace and improved accessions, while no obviously genetic diversity difference existed between landrace and improved accessions. The gene exchange between the landrace and improved accessions was more frequent than between the wild accession. A core germplasm collection was obtained, including 165 accessions (47 wild, 92 landrace, and 26 improved), with high genetic diversity and good representation. Our study provides an invaluable resource for the conservation of genetic diversity and the genetic improvement of Tartary buckwheat.

## Figures and Tables

**Table 1:** Characterization of genetic diversity via amplification using 15 SSR primer pairs in 659 Tartary buckwheat accessions.

No.	Marker	Na	Ne	Ho	He	PIC	I	Fis
1	SSR23	6	1.318	0.008	0.241	0.231	0.541	0.932
2	SSR36	9	1.546	0.071	0.353	0.319	0.693	0.817
3	Ft1ssR21	17	3.406	0.148	0.706	0.67	1.568	0.778
4	Ft3ssR15	6	1.811	0.343	0.448	0.422	0.912	0.300
5	Ft5ssR20	13	3.182	0.234	0.686	0.651	1.565	0.633
6	P128	18	1.770	0.110	0.435	0.424	1.134	0.729
7	SSRT26	7	1.018	0.015	0.018	0.018	0.072	0.310
8	SSRT33	4	1.900	0.691	0.474	0.377	0.727	−0.414
9	SSRT39	4	1.744	0.298	0.427	0.341	0.648	0.246
10	SSRT40	7	1.258	0.104	0.205	0.193	0.447	0.503
11	SWUFt169	5	1.476	0.03	0.323	0.296	0.636	0.895
12	SXAU2177	13	1.749	0.082	0.428	0.401	0.97	0.823
13	SXAU6249	13	1.813	0.082	0.448	0.434	1.088	0.827
14	TatG0052	12	2.431	0.160	0.589	0.559	1.336	0.713
15	TatG0200	8	2.537	0.982	0.606	0.529	1.091	−0.616
Total	-	142	28.959	3.358	6.387	5.865	13.428	7.476
Average	-	9.47	1.931	0.224	0.426	0.391	0.895	0.498

Notes: Na: number of alleles; Ne: effective number of alleles; Ho: observed heterozygosity; He: expected heterozygosity; PIC: polymorphic information content; I: Shannon’s information index; Fis: inbreeding coefficient.

**Table 2:** Characterization of genetic diversity among wild, landrace, and improved Tartary buckwheat groups.

Group	Na		Ne		Ho		uHe		I	
	Total	Average	Total	Average	Total	Average	Total	Average	Total	Average
Wild accession	107	7.133 ^a^	49.852	3.324 ^a^	3.371	0.225 ^a^	8.819	0.591 ^a^	18.520	1.23 ^a^
Landrace accession	115	7.667 ^a^	26.218	1.748 ^b^	3.413	0.228 ^a^	5.521	0.369 ^b^	10.966	0.731 ^b^
Improved accession	66	4.400 ^b^	24.847	1.657 ^b^	3.234	0.216 ^a^	4.865	0.326 ^b^	9.069	0.605 ^b^

Notes: Na: number of alleles; Ne: effective number of alleles; Ho: observed heterozygosity; uHe: unbiased expected heterozygosity; I: Shannon’s information index. Different letters for the average value represent the significant difference at *p* < 0.01.

**Table 3:** Gene flow (up triangle) and genetic differentiation (lower triangle) among wild, landrace, and improved accessions.

Accession	Wild Accession	Landrace Accession	Improved Accession
Wild accession	-	2.187	1.843
Landrace accession	0.103	-	33.908
Improved accession	0.119	0.007	-

**Table 4:** Genetic distance (up triangle) and genetic identity (lower triangle) among wild, landrace, and improved accessions.

Accession	Wild Accession	Landrace Accession	Improved Accession
Wild accession	-	0.256	0.281
Landrace accession	0.774	-	0.006
Improved accession	0.755	0.994	-

**Table 5:** Evaluation parameters of each primary core collection.

Population	*n*	Number of Polymorphic Loci	Percentage of Polymorphic Loci (%)	Na	Ne	Ho	uHe	I
All collection	659	142	100	9.467	1.931	0.224	0.426	0.895
30% Pre-core collection	198	135	95.07	9.000	2.443	0.288	0.527	1.123
25% Pre-core collection	165	131	92.25	8.733	2.561	0.297	0.541	1.158
20% Pre-core collection	132	131	92.25	8.733	2.689	0.298	0.559	1.205
15% Pre-core collection	99	130	91.55	8.677	2.775	0.305	0.570	1.233
10% Pre-core collection	66	122	85.92	8.133	3.140	0.311	0.602	1.307
8% Pre-core collection	53	116	81.69	7.733	3.334	0.324	0.610	1.324
t1				0.296	−1.666	−0.674	−1.456	−1.351
t2				0.470	−1.896	−0.764	−1.648	−1.529
t3				0.472	−2.190 *	−0.786	−1.892	−1.774
t4				0.516	−2.385 *	−0.858	−2.048	−1.918
t5				0.853	−2.811 **	−0.920	−2.431 *	−2.229 *
t6				1.170	−2.913 **	−0.622	−2.525 *	−2.326 *

Notes: n: number of samples; Na: number of alleles; Ne: effective number of alleles; Ho: observed heterozygosity; uHe: unbiased expected heterozygosity; I: Shannon’s information index. t1, t2, t3, t4, t5, and t6 represent the *t*-test value of genetic parameters of core collection (30%, 25%, 20%, 15%, 10%, 8% pre-core col-lection) and original collection, respectively; t (28, 0.05) = 2.048. * and ** represent the significant difference at *p* < 0.05 and *p* < 0.01, respectively.

**Table 6:** Comparison of genetic diversity among Tartary buckwheat core collection, original collection, and reserved collection.

Population	n	Na	Ne	Ho	uHe	I
All collection	659 (100%)	9.467	1.931	0.224	0.426	0.895
Core collection	165 (25.04%)	8.733	2.561	0.297	0.541	1.158
Reservation collection	494 (74.96%)	7.467	1.781	0.201	0.383	0.78
t1		0.470	−1.896	−0.764	−1.648	−1.529
t2		1.307	0.635	0.226	0.642	0.788

Notes: n: number of samples; Na: number of alleles; Ne: effective number of alleles; Ho: observed heterozygosity; uHe: unbiased expected heterozygosity; I: Shannon’s information index. t1 represents the *t*-test value of the genetic parameters of the core collection and the original collection, and t2 represents the *t*-test value of the genetic parameters of the reserved collection and the original collection; t (28, 0.05) = 2.048.

## Data Availability

All data are provided in the article. All the materials in this study are available upon request.

## Supplementary Materials

The following supporting information can be downloaded at https://www.mdpi.com/article/10.3390/plants14050771/s1: Table S1: Information on population structure of 659 Tartary buckwheat accessions; Table S2: Genetic distance results for 659 Tartary buckwheat accessions; Table S3: The (0, 1) matrix of 659 Tartary buckwheat accessions from 15 SSR markers; Table S4: Information on each subgroup from the UPGMA of 659 Tartary buckwheat accessions; Table S5: Information on core germplasm resources of the Tartary buckwheat; Table S6: Information on 15 SSR primers.
